# Mouse mammary tumour virus (MMTV) and human breast cancer with neuroendocrine differentiation

**DOI:** 10.1186/s13027-017-0135-8

**Published:** 2017-05-16

**Authors:** Lawson JS, Ngan CC, Glenn WK, Tran DD

**Affiliations:** 1 School of Biotechnology and Biomolecular Sciences, University of New South Wales, Sydney, Australia; 2 Douglass Hanly Moir Pathology, Sydney, Australia

**Keywords:** Breast cancer, Mouse mammary tumours, Mouse mammary tumour virus, Neuroendocrine breast cancer, Synaptophysin, Chromogranin

## Abstract

**Background:**

Mouse mammary tumour viruses (MMTVs) may have a role in a subset of human breast cancers. MMTV positive human breast cancers have similar histological characteristics to neuroendocrine breast cancers and to MMTV positive mouse mammary tumours. The purpose of this study was to investigate the expression of neuroendocrine biomarkers – synaptophysin and chromogranin, to determine if these histological characteristics and biomarker expression were due to the influences of MMTV.

**Methods:**

Immunohistochemistry analyses to identify synaptophysin and chromogranin were conducted on a series of human breast cancers in which (i) MMTV had been previously identified and had similar histological characteristics to MMTV positive mouse mammary tumours and (ii) MMTV positive mouse mammary tumours.

**Results:**

The expression of synaptophysin and chromogranin in MMTV positive mouse mammary tumors were all positive (7 of 7 specimens – 100% positive). The expression of synaptophysin and chromogranin in MMTV positive human breast cancers was much less prevalent (3 of 22 – 14%). There was no expression of synaptophysin and chromogranin in the normal breast tissue control specimens.

**Discussion:**

It is not possible to draw any firm conclusions from these observations. However, despite the small numbers of MMTV positive mouse mammary tumours in this study, the universal expression in these specimens of synaptophysin and chromogranin proteins is striking. This pattern of synaptophysin and chromogranin expression is very different from their expression in MMTV positive human breast cancers. The reason for these differences is not known.

**Conclusions:**

The high prevalence of positive expression of synaptophysin and chromogranin in MMTV positive mouse mammary tumours and low expression of synaptophysin and chromogranin in MMTV positive human breast cancers indicates that MMTV is not usually associated with neuroendocrine human breast cancers.

**Electronic supplementary material:**

The online version of this article (doi:10.1186/s13027-017-0135-8) contains supplementary material, which is available to authorized users.

## Background

There is substantial, but not conclusive, evidence that MMTVs may have a role in a subset of human breast cancers. This evidence is as follows: (i) identification of MMTV–like gene sequences in breast cancer tissues is associated with a 15 fold increase in breast cancer [1], (ii) MMTV-like *env* gene sequences have been identified in 38% of US and Australian human breast tumours but rarely in normal breast tissue controls [2, 3], (iii) MMTV sequences identified in human breast tissues are 95 to 98% homologous with MMTV in mouse mammary tumours [4, 5], (iv) MMTV viral proteins have been identified in human breast cancer [6, 7], (v) Wnt-1 oncogene expression is significantly higher in MMTV-like positive compared to MMTV-like negative breast cancer specimens, which parallels high Wnt-1 expression in MMTV positive mouse mammary tumours [8], (vi) MMTV can infect human cells and randomly integrate its genomic information [9–11], (vii) there is increased prevalence of MMTV-like viral sequences in healthy breast tissues (nil), healthy tissue adjacent to breast cancer (19%), breast hyperplasia (27%), ductal carcinoma in situ (82%) [12], (viii) MMTV–like sequences have been identified in milk from healthy lactating women and three fold increased positivity in milk from women at high risk for breast cancer [13, 14], (ix) MMTV-like sequences have been identified in the saliva of 27% of healthy children, 11% of healthy adults and 57% of adults with breast cancer, which is suggestive of a human to human viral transmission [15], (xi) MMTV-like viral sequences have been identified in breast cancers which developed in a father, mother and daughter of the same family which is suggestive of an infectious condition [16] and MMTV sequences have been identified in benign human breast tissues before the development of MMTV associated breast cancer in the same women [17]. Overall this evidence is consistent with MMTV having similar influences in both human breast cancer and mouse mammary tumours.

We have previously observed that the histological characteristics of MMTV positive human breast cancers are similar to MMTV associated mouse mammary tumours [18]. Many of these MMTV positive human breast cancers also have similar histology to neuroendocrine human breast cancers. Neuroendocrine breast cancer is diagnosed by both histological characteristics and the expression of either synaptophysin or chromogranin proteins [19]. Initially, the World Health Organisation(WHO) classified breast cancers with neuroendocrine features as those breast tumours with over 50% of positive synaptophysin or chromogranin cancer cells. This has since been modified to include breast cancers with any number of synaptophysin or chromogranin positive cancer cells. The WHO classsification now refers to such breast tumours as invasive breast carcinomas with neuroendocrine differentiation [20]. Synaptophysin and chromogranin are proteins secreted by endocrine (hormone producing) cells located in many organs of the body in response to a ‘neural’ (brain or nervous system) stimulus. While synaptophysin and chromogranin proteins may be secreted by both normal and cancer endocrine cells, their expression is usually higher in malignant cells. This phenomena can be used for diagnostic purposes [21].

In a preliminary investigation we observed that synaptophysin or chromogranin were highly expressed in MMTV associated mouse mammary tumours. As synaptophysin and chromogranin proteins are expressed in human breast cancers which have similar histological characteristics to MMTV positive mouse mammary tumours, we hypothesised that MMTV may be the underlying causal factor.

To explore this hypothesis we investigated by immunohistochemistry, the expression of synaptophysin and chromogranin in a series of MMTV positive human breast cancers and MMTV positive mouse mammary tumours. We have not been able to identify any prior investigations into the expression of synaptophysin and chromogranin in MMTV positive human breast cancers or MMTV positive mouse mammary tumours.

Here we show that the expression of synaptophysin and chromogranin in MMTV positive breast cancers is not usually associated with human neuroendocrine breast cancers.

## Methods

### Ethics

This project was approved by the Human Research Ethics Committee of the University of New South Wales, Sydney, Australia (HC11421).

### Human breast specimens

Twenty breast cancer archival formalin fixed breast cancer specimens were selected because in previous studies MMTV envelope gene sequences had been identified in these specimens [3]. The MMTV sequences had been identified by PCR techniques following the methods of Wang et al. [2]. In addition each of these breast cancer specimens were selected because their histological characteristics were similar to MMTV positive mouse mammary tumours.

Thirty eight normal breast specimens from breast reduction surgery were used as controls. The donors of these specimens were on average younger than the breast cancer patients and therefore should be considered as a comparison group and not age matched controls.

### Mouse mammary tumour specimens

Seven mouse mammary tumours in which MMTV envelope gene sequences had previously been identified were used for comparative purposes [22].

### Immunohistochemistry

The automated Tissue-Tek Prisma system were used for haematoxylin and eosin staining and Ventana BenchMark Ultra were used for the identification of synaptophysin and Chromogranin A proteins. Synaptophysin (Novocastra catalogue number NCL-L-SYNAP-299) and chromogranin A (Dako catalogue number M0869) antibodies were used on formalin fixed paraffin embedded specimens. Both synaptophysin and chromogranin A proteins stain the cell membranes and cytoplasm of the target cells. Pancreatic tissues were used as positive controls for each specimen. Omission of the antibodies was used as negative controls.

### Statistics

The non-parametric Kolmogorov-Smirnov test was used to test the significance between the human breast cancer and mouse mammary tumour synaptophysin and chromogranin positivity.

## Results

### Synaptophysin and chromogranin expression

The outcomes are presented in Table 1 and Additional file 1: Table S1, Additional file 2: Table S2 and Additional file 3: Table S3. The expression of synaptophysin and chromogranin in MMTV positive mouse mammary tumours were all positive (7 of 7 specimens – 100% positive). The expression of synaptophysin and chromogranin in MMTV positive human breast cancers was much less prevalent (3 of 22 – 14%). There was no expression of synaptophysin and chromogranin in the normal breast tissue control specimens. The different positivity for synaptophysin and chromogranin between the human breast cancer and mouse mammary tumour was p = 0.001 and 0.001 respectively, that is highly significant.

**Table 1:** Synaptophysin and chromogranin protein expression in MMTV positive mouse mammary tumors, MMTV positive human breast cancers and normal human breast controls

Specimens	MMTV	Synaptophysin	Chromogranin
Mouse mammary tumour	7/7 (100%)	7/7 (100%)	7/7 (100%)
Human breast cancer (selected for MMTV positivity)	22/22 (100%)	2/22 (9%)	2/22 (9%)
Normal human breast controls	0/39 (0%)	0/39 (0%)	0/39 (0%)

**Fig. 1:**
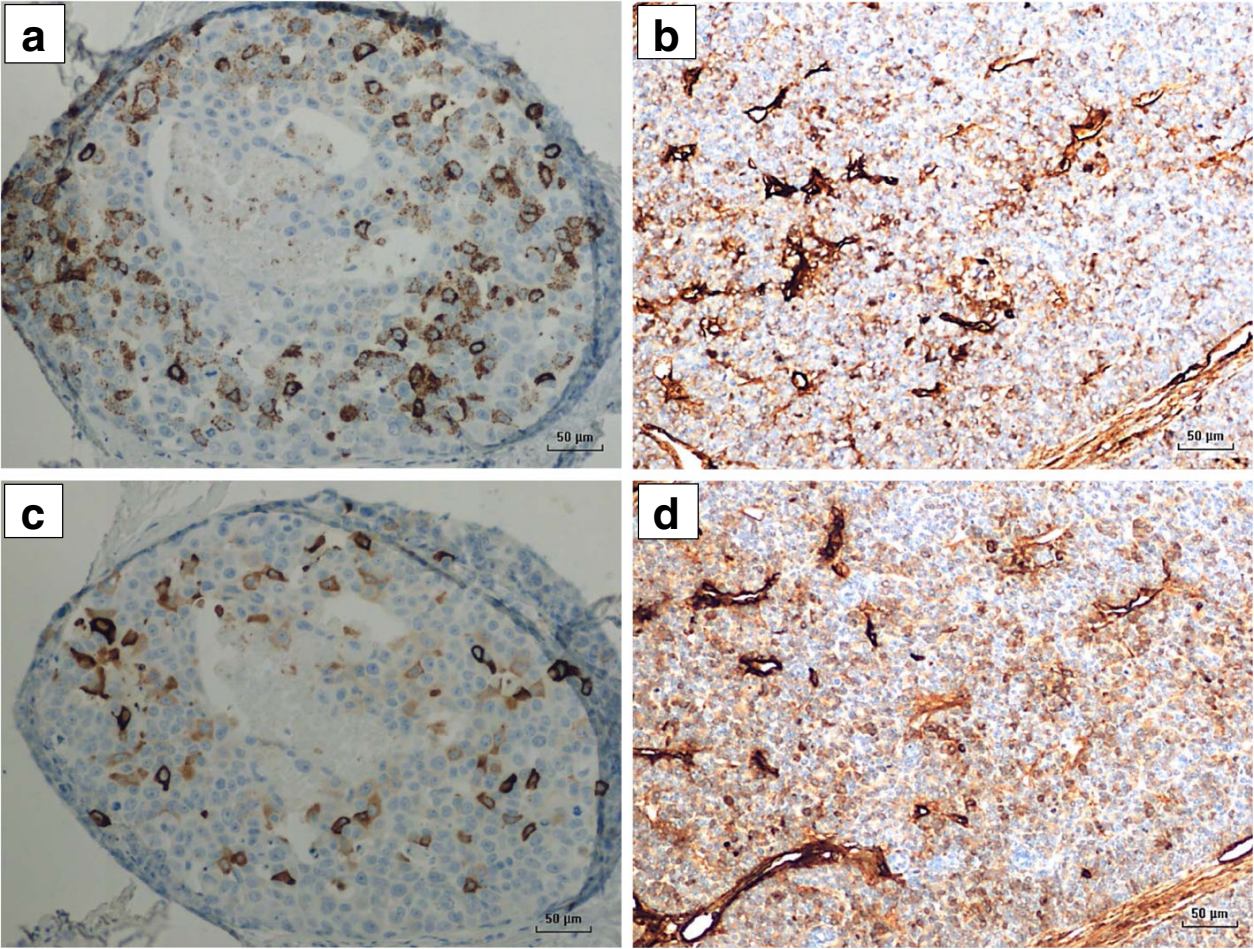
Synaptophysin & Chromogranin protein expression in MMTV positive human breast cancer & MMTV positive mouse mammary tumour. **a**. Human synaptophysin expression. **b**. Mouse synaptophysin expression. **c**. Human chromogranin expression. **d**. Mouse chromogranin expression

The expression of synaptophysin and chromogranin in MMTV positive mouse mammary tumours and MMTV positive human breast cancers are shown in Fig. 1.

### Histology

**Fig. 2:**
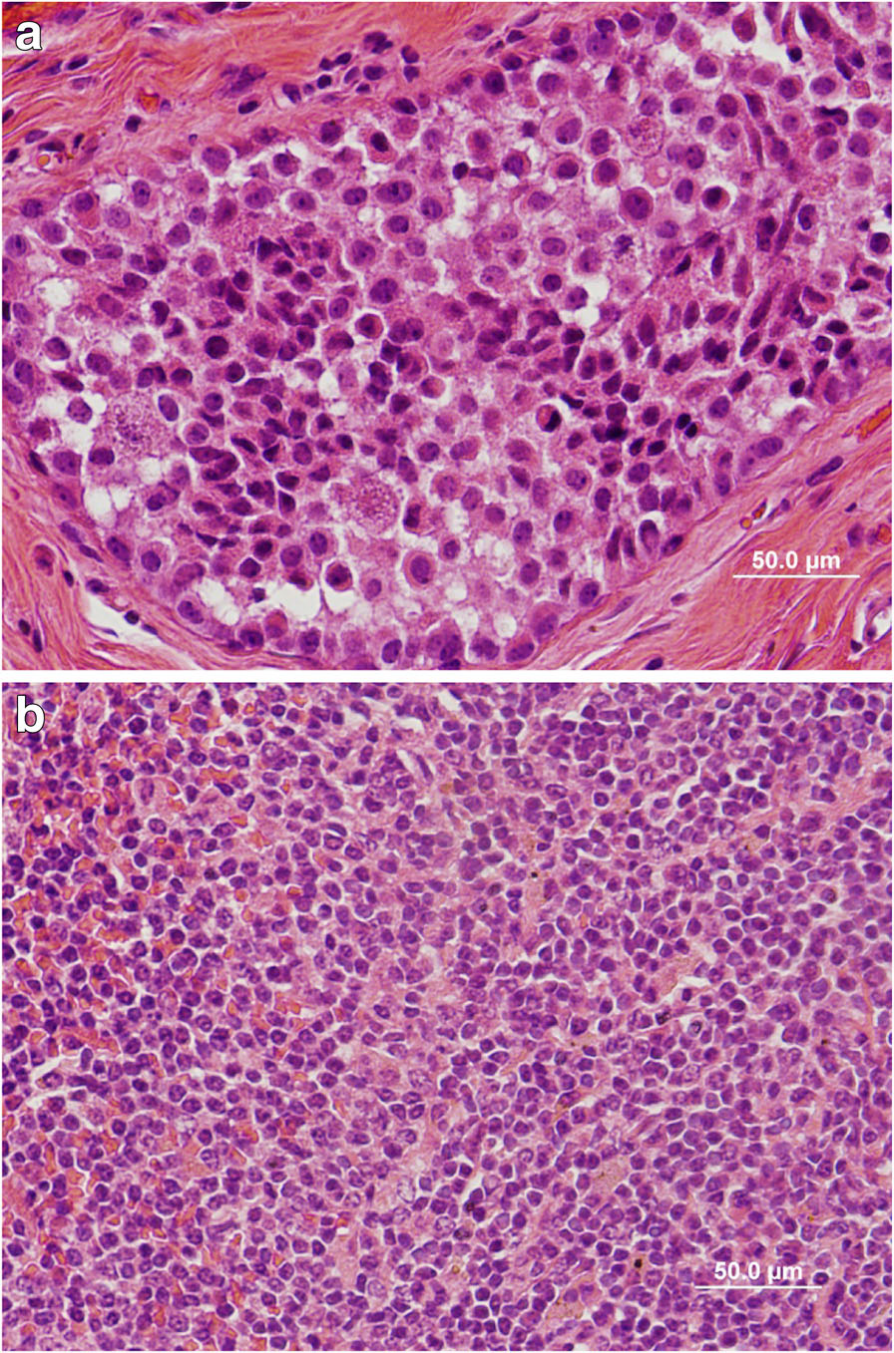
**a**. MMTV positive human breast cancer. **b**. MMTV positive Dunn type B mouse mammary tumour (Haematoxylin and eosin stains)

The similar histological characteristics of MMTV positive and neuroendocrine marker positive human breast cancers and MMTV neuroendocrine positive mouse mammary tumours are shown in Fig. 2. Mouse mammary tumour cells are smaller in diameter than human breast cancer cells but have very similar characteristics. However it must be emphasised that the histological characteristics of MMTV positive and synaptophysin and chromogranin *negative* human breast cancers are also similar to MMTV positive mouse mammary tumours. The synaptophysin and chromogranin positive MMTV positive human breast cancers are characterised by intensely stained nuclei which occupy most of the cell, the cells are mostly round and regular in size and are clumped together without glandular acini or lumen. MMTV negative human breast cancers are not similar to MMTV positive mouse mammary tumours.

**Fig. 3:**
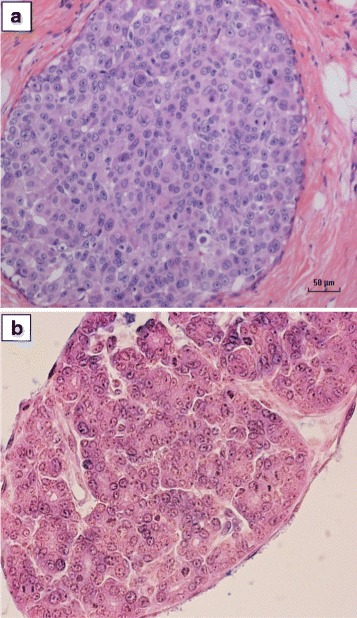
Breast cancer specimen from patient 8 which was positive for both Synaptophysin & Chromogranin expression. **a**. MMTV positive human breast cancer. **b**. MMTV positive Dunn type B mouse mammary tumour (Haematoxylin and eosin stains)

One human breast cancer specimen was positive for both Synaptophysin and Chromogranin. As shown in Fig. 3 the histological characteristics of this specimen were similar to MMTV positive mouse mammary tumours.

## Discussion

We have demonstrated that (i) MMTV positive mouse mammary tumours are all synaptophysin and chromogranin positive (7 of 7 specimens – 100% positive), (ii) in MMTV positive human breast cancers only 3 of 22 (14%) are synaptophysin or chromogranin positive, (iii) there was no expression of synaptophysin and chromogranin in the comparative normal breast specimens and (iv) MMTV positive (and either synaptophysin and chromogranin positive or negative) human breast cancers have similar histological characteristics to neuroendocrine human breast cancers and to MMTV positive mouse mammary tumours.

The expression of synaptophysin and chromogranin proteins in MMTV positive human breast cancers was much less prevalent than in MMTV positive mouse mammary tumours.

These are confusing observations and it is not possible to draw any firm conclusions. However, despite the small numbers of mouse mammary tumours in this study, the universal expression in these specimens of synaptophysin and chromogranin proteins is striking. This pattern of synaptophysin and chromogranin expression is very different from the MMTV human breast cancers. The reason is not known.

The expression or secretion of synaptophysin and chromogranin proteins by endocrine (hormone) producing cells is presumably in response to a neurological or other external stimulus. However, the substantial differences in the expression of synaptophysin and chromogranin in MMTV positive human breast cancer and MMTV positive mouse mammary tumours indicate that MMTV is not usually associated with neuroendocrine human breast cancers.

It has been suggested by Wiedenmann et al. [21] and later by Maeda et al. [23] that the identification of synaptophysin may be useful for the diagnosis of breast cancers. The findings in this current study do not support that suggestion.

## Conclusions

The high prevalence of positive expression of synaptophysin and chromogranin in MMTV positive mouse mammary tumours and low expression of synaptophysin and chromogranin in MMTV positive human breast cancers indicates that MMTV is not usually associated with neuroendocrine human breast cancers.

## Additional files

Additional file 1: Table S1. Selected MMTV positive human breast cancers. Synaptophysin and chromogranin expression. (XLSX 11 kb)

Additional file 2: Table S2. Mouse mammary tumours. Synaptophysin and chromogranin expression (XLSX 10 kb)

Additional file 3: Table S3. Normal human breast specimens. MMTV negative. Synaptophysin and chromogranin (XLSX 11 kb)

## Abbreviation

MMTV: Mouse mammary tumor virus
