# Correction to: mouse mammary tumour virus (MMTV) and human breast cancer with neuroendocrine differentiation

**DOI:** 10.1186/s13027-017-0161-6

**Published:** 2017-10-06

**Authors:** J. S. Lawson, C. C. Ngan, W. K. Glenn, D. D. Tran

**Affiliations:** 1 School of Biotechnology and Biomolecular Sciences, University of New South Wales, Sydney, Australia; 2 Douglass Hanly Moir Pathology, Sydney, Australia

After publication of the original article [1], the authors realised their given names and family names were incorrectly transposed in the author list. The correct presentations of the authors’ names are included in the author list of this correction article.
